# Identifying genetic hypomethylation and upregulation of toll-like receptors in Kawasaki disease

**DOI:** 10.18632/oncotarget.14497

**Published:** 2017-01-04

**Authors:** Ying-Hsien Huang, Sung-Chou Li, Lien-Hung Huang, Pao-Chun Chen, Yi-Yu Lin, Chiung-Chun Lin, Ho-Chang Kuo

**Affiliations:** ^1^ Department of Pediatrics, Kaohsiung Chang Gung Memorial Hospital and Chang Gung University College of Medicine, Kaohsiung, Taiwan; ^2^ Kawasaki Disease Center, Kaohsiung Chang Gung Memorial Hospital, Taiwan; ^3^ Genomics and Proteomics Core Laboratory, Department of Medical Research, Kaohsiung Chang Gung Memorial Hospital and Chang Gung University College of Medicine, Kaohsiung, Taiwan; ^4^ Department of Nursing, Kaohsiung Chang Gung Memorial Hospital

**Keywords:** toll-like receptor, IVIG, kawasaki disease, methylation

## Abstract

Kawasaki disease (KD) is an acute febrile systemic vasculitis that occurs in children and is characterized by elevated levels of proinflammatory cytokines. Toll-like receptors (TLRs) serve as the sensor arm of the innate immune system and induce proinflammatory cytokine expressions.

We recruited a total of 18 paired KD patients, before intravenous immunoglobulin (IVIG) and at least 3 weeks after IVIG treatment, 18 healthy controls, and 18 febrile controls. For TLR genes and their cytosine-phosphate-guanine (CpG) markers, we used Affymetrix GeneChip^®^ Human Transcriptome Array 2.0 and Illumina HumanMethylation450 BeadChip to evaluate gene expression levels and methylation patterns, respectively.

KD patients demonstrated a significantly differential expression of TLR mRNA levels compared to both the healthy and febrile controls, with only TLR 3 and 7 not differing between the KD patients and the controls. After patients underwent IVIG treatment, the TLR mRNA levels, except for TLR3, decreased significantly in KD patients. In contrast, the methylation status of the CpG sites of TLR1, 2, 4, 6, 8, and 9 demonstrated an opposite tendency between the two stages of both the KD samples and the controls.

TLRs, particularly TLR1, 2, 4, 6, 8, and 9, may stimulate the immunopathogenesis of KD. These results are among the first to use TLRs to prove that a bacterial inflammatory response may trigger KD.

## INTRODUCTION

Kawasaki disease (KD) is a form of acute vasculitis syndrome that impacts various systems. Although its etiology is still unknown, the disease most commonly occurs in children under the age of 5 years old [1]. The most serious complications of KD are coronary artery lesions (CAL), myocardial infarction, and coronary artery aneurysm formation [2].

The receptors on the host cells acting at the host-pathogen interface as the first surveillance system for primary infection are called pattern recognition receptors (PRRs). The family of toll-like receptors (TLRs) is the best characterized class of PRRs in mammalian species [3]. TLR signaling can result in either the production of type 1 interferon co-stimulatory molecules or the induction of a proinflammatory cytokine cascade [4], which includes various known cytokines and chemokines, such as TNF-α, IL-6, IL-8, MCP-1, and antimicrobial peptides, all of which have elevated values in the acute stage plasma of KD [5–7]. Currently, 10 TLRs have been identified in humans and 12 in mice, including bacterial peptidoglycan (detected by TLR1), lipoprotein and lipoteichoic acids (detected by TLR2), double-stranded RNA (detected by TLR3), LPS (detected by TLR4), flagellin (detected by TLR5), single-stranded viral RNA (detected by TLR7 and 8), and the unmethylated CpG (cytosine-phosphate-guanine) DNA of bacteria and viruses (detected by TLR9) [8]. Although researchers have yet to unequivocally identify consistent infectious pathogens in KD patients [9, 10], we previously found elevated TLR2 expression on monocytes in both KD patients and mice with lactobacillus casei cell wall extract (LCWE)-induced coronary arteritis [5]. Furthermore, previous studies have shown that TLR 2, 3, 4, 6, and 9 may trigger the initial immune response in KD patients [11–13].

The DNA methylation alteration of CpG sites indicates the reversal change in gene expression [14]. In a recent study, we reported a significant correlation between the promoter methylation of gamma Fc region receptor II-a among those susceptible to KD and the clinical outcomes of intravenous immunoglobulin (IVIG) administration. Furthermore, IVIG administration considerably increases methylation in KD [15]. Therefore, studying the differential expressions and methylation alteration of TLRs between KD patients and control subjects has merit. No studies have yet surveyed TLRs 1–10 in the same report or performed analysis with methylation profile. Therefore, in this study, we examine mRNA expression of TLRs 1-10 and perform analysis with methylation change in two different stages of KD patients and controls.

## RESULTS

### Differential expression of TLR mRNA levels among KD patients and controls

**Figure 1:**
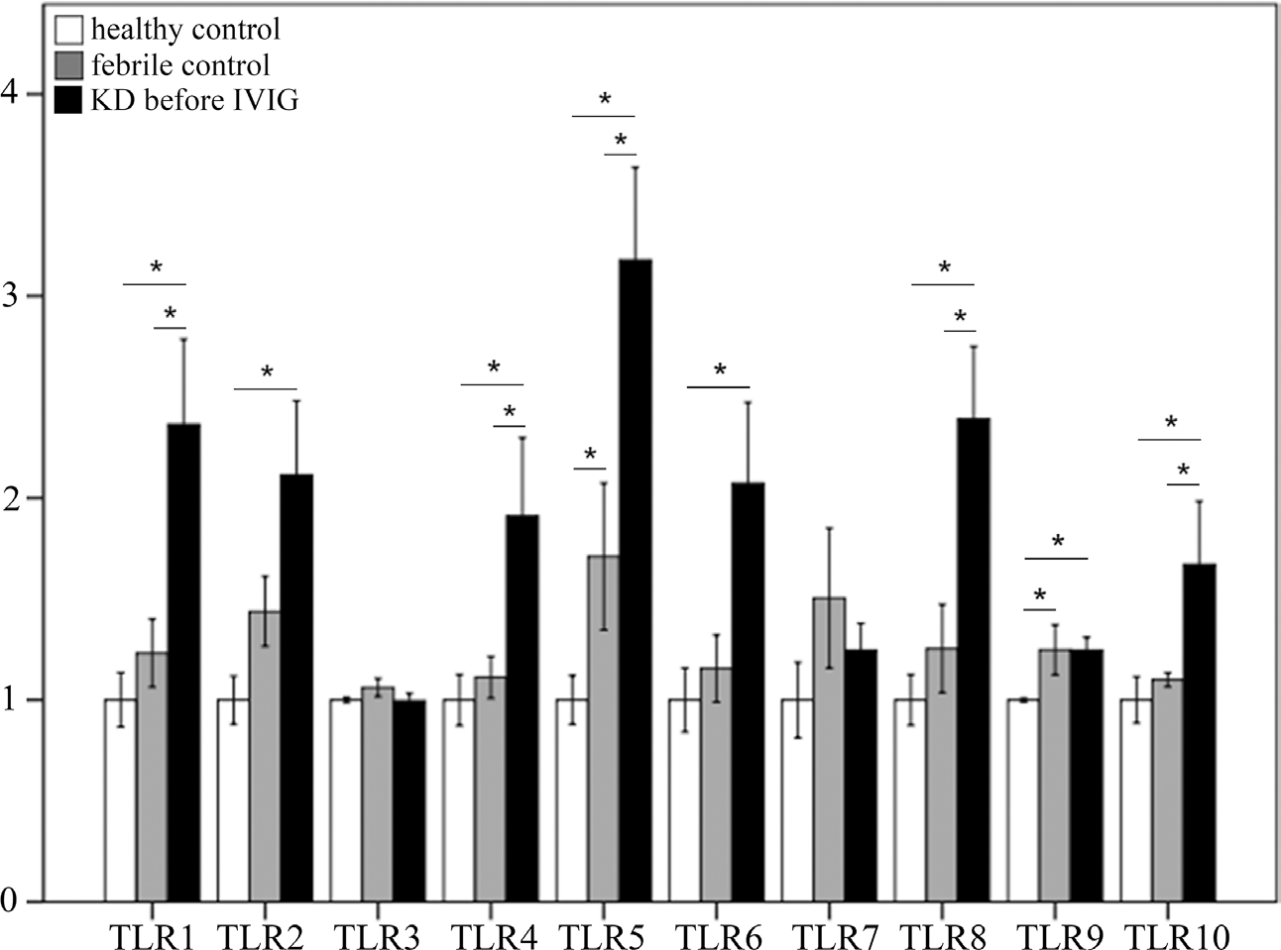
Comparison of toll-like receptors’ (TLRs) mRNA expressions by geneChip^®^ human transcriptome array 2.0 between acute-stage Kawasaki disease patients and control subjects Asterisks denote significance (*p* < 0.05). Data are expressed as mean ± standard error for the three replications.

KD patients showed differential expression of TLRs when compared to the healthy and febrile control subjects. The mRNA levels of TLR1, 4, 5, 8, and 10 were significantly elevated in KD patients compared to both the healthy control and febrile control groups (Figure 1). The mRNA levels of TLR2, 6, and 9 were significantly elevated in the KD patient group versus the healthy controls (Figure 1), while the mRNA levels of TLR 3 and 7 did not differ significantly among the groups.

### Decreased expression of TLRs’ mRNA levels among KD patients following IVIG administration

**Figure 2:**
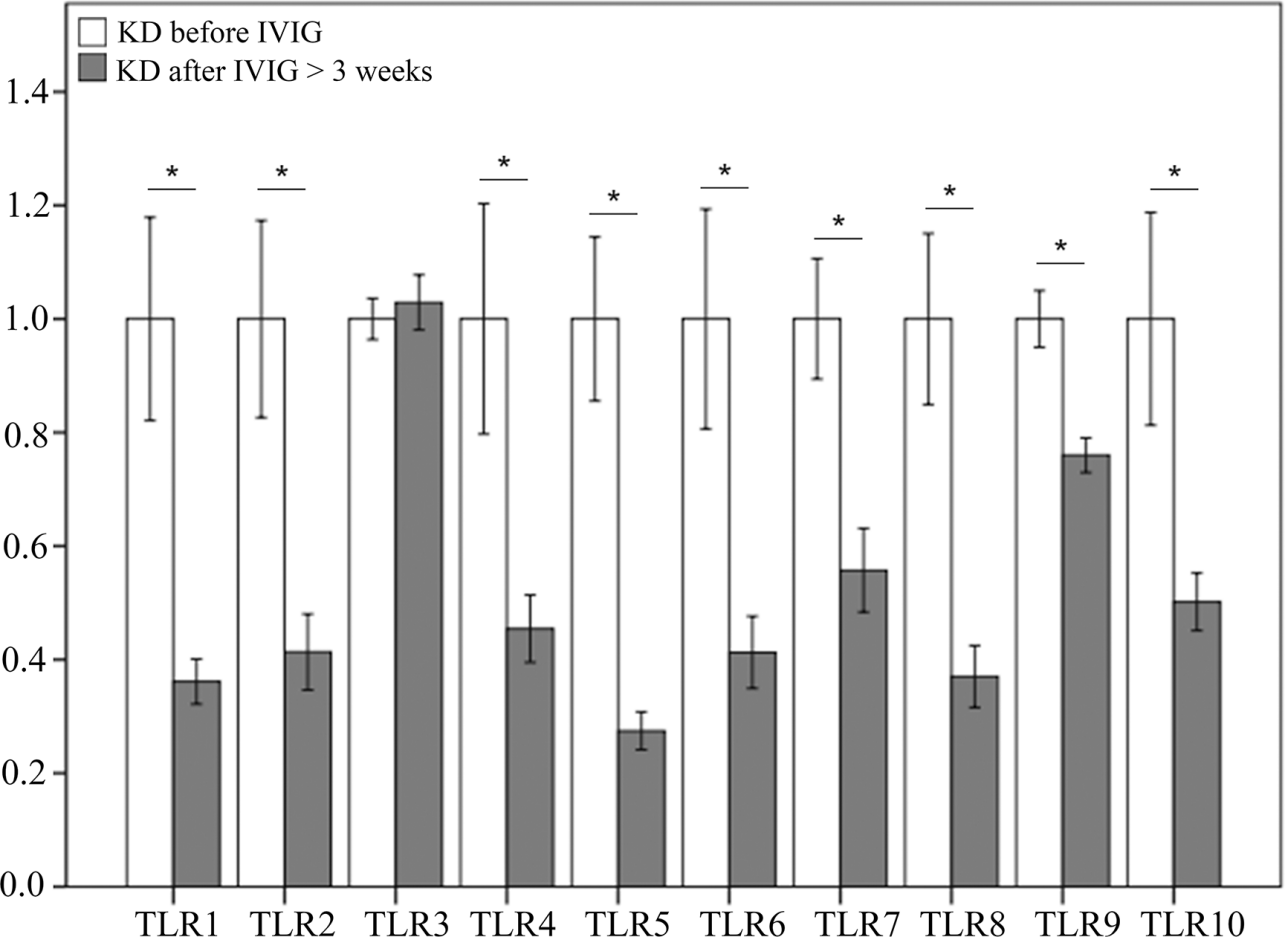
Comparison of toll-like receptors’ (TLRs) mRNA expressions by GeneChip^®^ human transcriptome array 2.0 between acute-stage Kawasaki disease patients and KD patients after receiving IVIG treatment Asterisks denote significance (*p* < 0.05). Data are expressed as mean ± standard error for the three replications.

As shown in Figure 2, the mRNA levels of TLR1, 2, 4, 5, 6, 7, 8, 9, and 10 decreased significantly in KD patients after undergoing IVIG treatment, while only the mRNA levels of TLR 3 did not differ significantly in KD patients following IVIG treatment.

### Differential expression of TLRs’ mRNA levels among KD patients after undergoing IVIG treatment and control subjects

**Figure 3:**
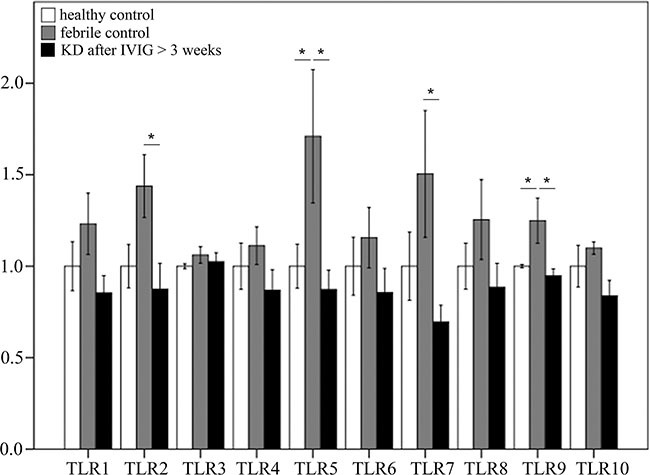
Comparison of toll-like receptors’ (TLRs) mRNA expressions by GeneChip^®^ human transcriptome array 2.0 between Kawasaki disease patients after IVIG treatment and control subjects Asterisks denote significance (*p* < 0.05). Data are expressed as mean ± standard error for the three replications.

As shown in Figure 3, we found no notable differences in the mRNA levels of TLRs between KD patients that received IVIG treatment and the healthy controls. The mRNA levels of TLR2, 5, 7, and 9 were significantly higher in the febrile controls than in the KD patients after receiving IVIG treatment (Figure 3).

### Significantly altered CpG sites on TLRs between KD patients and controls

**Figure 4:**
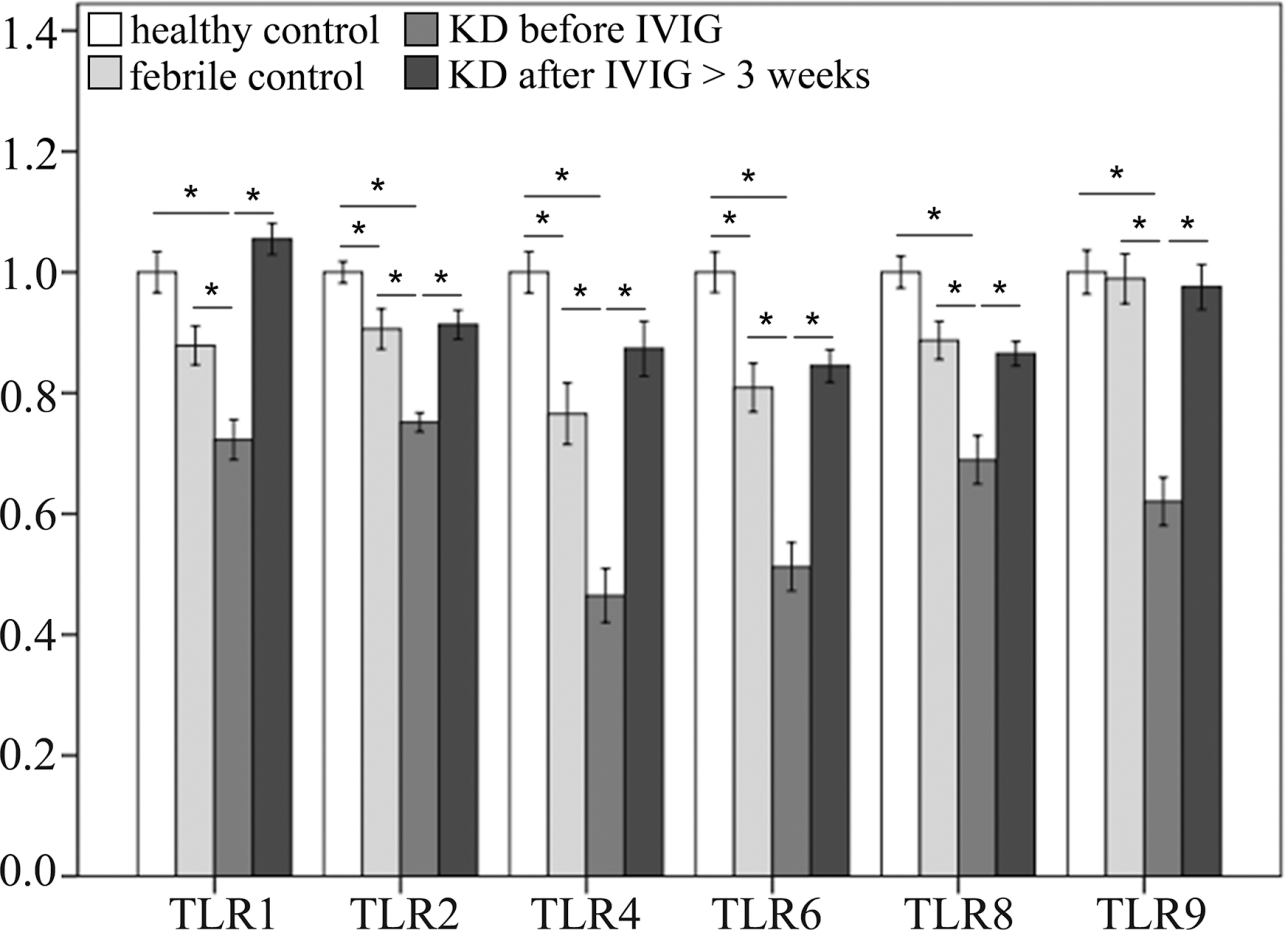
Comparison of methylation patterns of CpG markers on toll-like receptors (TLRs) A CpG marker was defined to be on a gene when located within the −5000 to +3000 region of a gene. Asterisks denote significance (*p* < 0.05). Data are expressed as mean ± standard error for the 12 replications.

**Figure 5:**
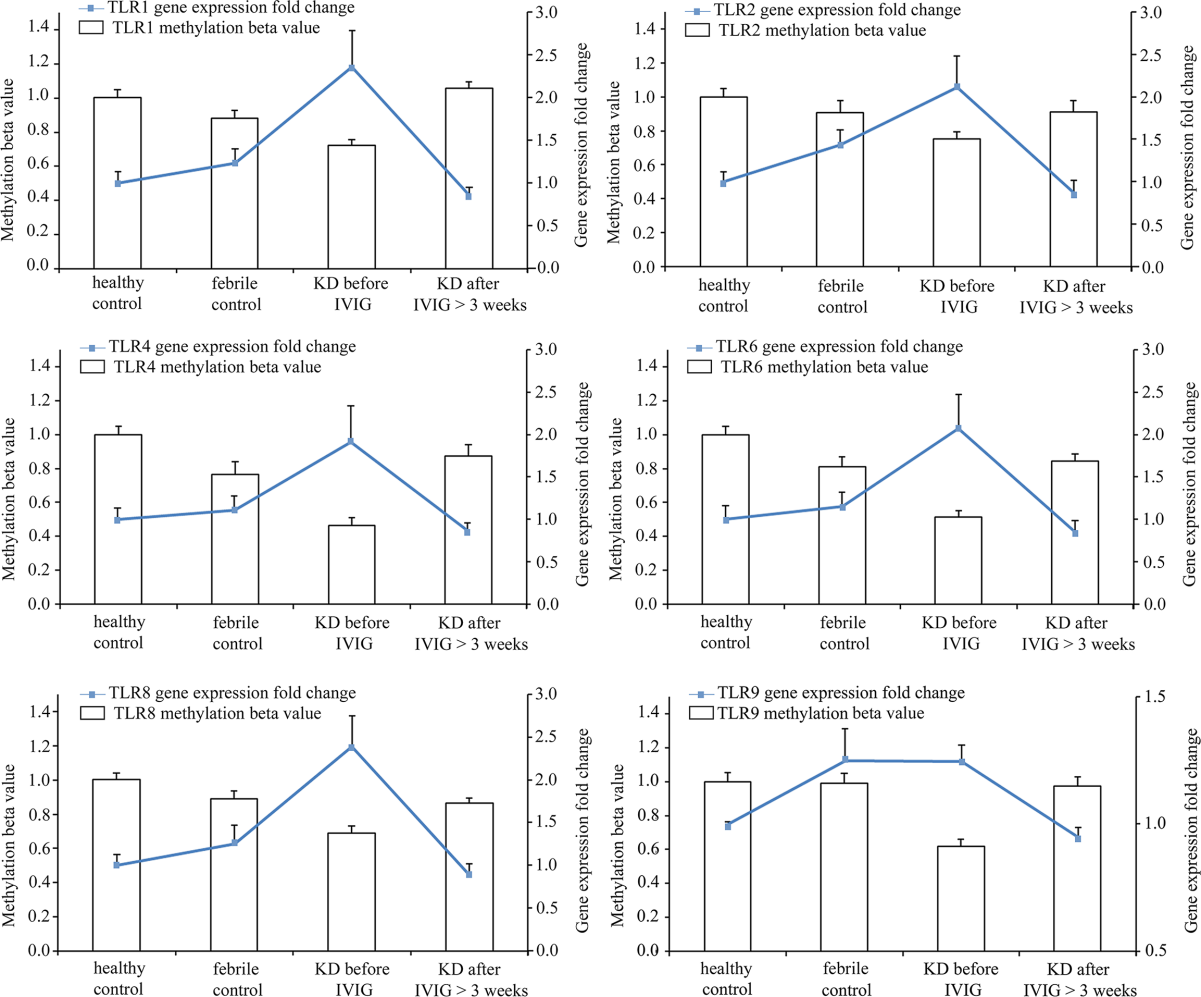
Integration of CpG marker methylation pattern and gene expression profile The CpG markers corresponding to TLR1, 2, 4, 6, 8, and 9 are cg22839308, cg03523945, cg13730105, cg14578677, cg07759587, and cg21578541, respectively. The methylation patterns of representative CpG markers and gene expression profiles of TLRs showed negative tendencies. Furthermore, they were found to be altered in the healthy and febrile control subjects, as well as in Kawasaki disease patients before and after IVIG treatment. For one TLR, the fold change values of gene expression in groups were determined by dividing them by the average of normal controls. The histogram and curve are presented as mean ± standard error.

**Figure 6:**
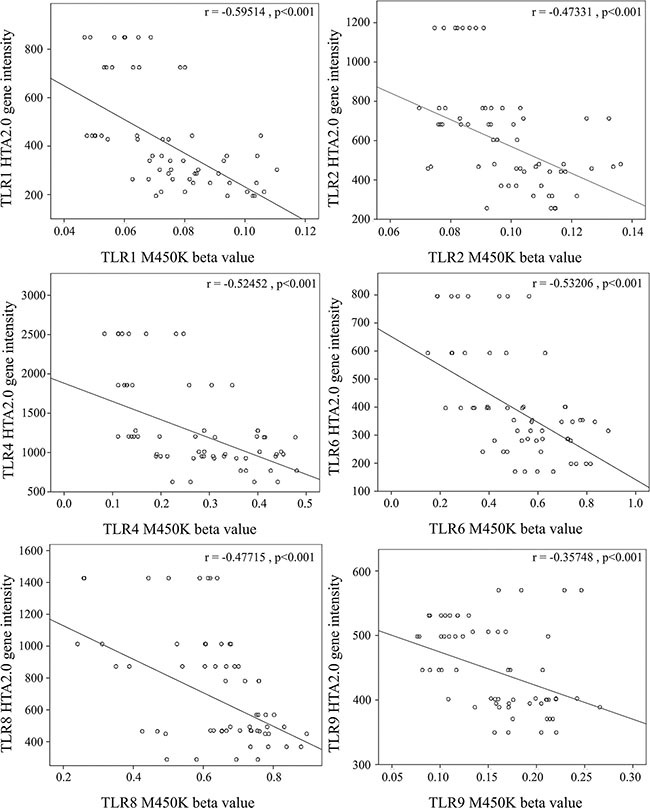
Correlation analysis of gene expression and CpG marker methylation The CpG markers corresponding to TLR1, 2, 4, 6, 8, and 9 are cg22839308, cg03523945, cg13730105, cg14578677, cg07759587, and cg21578541, respectively. We used scatter plots to demonstrate correlations between mRNA levels and DNA methylation. The results demonstrated that mRNA levels were negatively correlated with DNA methylation (Pearson's correlation coefficient around −0.4 and *p* < 0.001).

In this study, we used Infinium HumanMethylation450 BeadChip (Illumina) to evaluate methylation patterns of CpG sites on TLRs between KD patients and control subjects and found significantly altered CpG sites on TLRs (Table 1). We found that the methylation levels of TLR1, 2, 4, 6, 8, and 9 were significantly decreased in the acute stage of KD patients compared to the healthy and febrile controls (Figure 4). However, after IVIG administration, the methylation status of TLR1, 2, 4, 6, 8, and 9 was significantly elevated in KD patients (Figure 5). Since lower methylation leads to greater gene expression [15], we focused on the correlation between DNA methylation patterns and gene expressions. As shown in Figure 5, TLR1, 2, 4, 6, 8, and 9 demonstrate a hypo-methylated status in KD patients prior to undergoing IVIG treatment compared with the control subjects and the KD patients after undergoing IVIG treatment. Therefore, the mRNA expression level and DNA methylation of these TLRs demonstrate negative correlations. To ensure un-biased results, we performed 10,000 re-samplings [16] for each TLR to extract pairs of gene intensity and CpG marker β values in each group. Therefore, we collected 40,000 pairs of gene intensity and CpG marker β values from the four groups. Figure 6 demonstrates that gene expression level and CpG marker methylation were significantly negatively correlated (*p* < 0.001 for all TRLs), thus indicating that DNA methylation represses gene expression.

**Table 1:** Methylation patterns of CpG sites on toll like receptors between Kawasaki disease patients and control subjects

Target ID	Symbol	Fold-Change (KD1 vs. HC)	*p*-value (KD1 vs. HC)	Fold-Change (KD1 vs. FC)	*p*-value (KD1 vs. FC)	Fold-Change (KD2 vs. KD1)	*p*-value (KD2 vs. KD1)
cg02016764	TLR1	1.01476	0.368	1.05872	0.001*	1.01892	0.251
cg08757862		−1.01801	7.79E-06*	1.0037	0.312	1.00642	0.083
cg09316306		−1.05901	2.06E-09*	−1.01487	0.071	1.06207	5.26E-10*
cg22839308		−1.01643	6.46E-06*	−1.00921	0.007*	1.01972	1.73E-07*
cg02345613	TLR2	−1.00936	0.02*	−1.00508	0.197	−1.0018	0.646
cg03523945		−1.01976	4.84E-08*	−1.01223	0.000*	1.01281	0.000*
cg03610073		−1.0096	0.248	−1.00462	0.575	−1.02875	0.001*
cg06405222		−1.00235	0.397	1.00353	0.206	−1.01922	4.88E-09*
cg06618866		−1.0313	2.42E-06*	−1.02716	0.000*	1.00971	0.105
cg15852258		1.00144	0.571	1.00659	0.012*	1.0127	5.92E-06*
cg16547110		1.0028	0.259	1.00607	0.016*	−1.01118	3.00E-05*
cg17916835		−1.01637	0.007*	−1.00295	0.612	1.00299	0.608
cg19037167		−1.02192	2.37E-07*	−1.01582	0.000*	1.00948	0.013*
cg02821380	TLR3	−1.00465	0.913	1.00428	0.920	−1.03928	0.365
cg06498520		−1.0229	0.012*	−1.03399	0.000*	1.06263	4.44E-09*
cg12281049		−1.00299	0.256	−1.01884	0.000*	1.01653	4.83E-08*
cg23601030		1.00011	0.994	1.02091	0.151	−1.03026	0.04*
cg04061482	TLR4	1.00672	0.861	1.01445	0.707	−1.00803	0.834
cg05429895		−1.01334	1.57E-06*	−1.01838	0.000*	1.02266	1.35E-12*
cg13730105		−1.15383	5.19E-09*	−1.08378	0.000*	1.11546	2.31E-06*
cg00255925	TLR5	1.00094	0.774	1.01447	0.000*	−1.02364	1.63E-09*
cg01181681		1.05831	0.001*	1.03307	0.048*	−1.0815	9.88E-06*
cg03702975		1.0162	0.021*	−1.00727	0.290	−1.01852	0.009*
cg04219417		1.04379	0.002*	1.02063	0.131	−1.07478	1.36E-06*
cg05696109		−1.00085	0.742	−1.00095	0.703	1.01726	5.81E-09*
cg05858079		−1.01157	0.041*	−1.00032	0.954	−1.02197	0.000*
cg07015886		1.05173	0.001*	1.03711	0.012*	−1.09629	1.93E-08*
cg07538512		−1.00577	0.056	1.00421	0.159	1.01089	0.001*
cg07574686		−1.00294	0.515	−1.00419	0.355	−1.00194	0.667
cg09025215		1.04164	0.001*	1.02776	0.028*	−1.04867	0.000*
cg12275981		1.02507	0.001*	−1.00798	0.259	−1.01615	0.025*
cg12900151		−1.01145	0.608	−1.00222	0.920	−1.06584	0.006*
cg13557530		−1.00724	0.103	1.00232	0.598	−1.0567	4.69E-18*
cg14015211		−1.00305	0.238	1.00024	0.926	−1.02511	1.40E-13*
cg14228103		1.02911	0.512	1.02499	0.573	−1.08018	0.082
cg17599809		1.00068	0.568	1.00109	0.364	1.00403	0.001*
cg02221520	TLR6	−1.20594	7.72E-11*	−1.10954	0.000*	1.12684	4.16E-06*
cg04840108		−1.16201	1.24E-10*	−1.07016	0.001*	1.08012	0.000*
cg10182418		−1.02683	0.0004707*	−1.02678	0.000*	1.0643	4.70E-12*
cg14578677		−1.27206	3.13E-10*	−1.1577	0.000*	1.17843	2.84E-06*
cg14665413	TLR6	−1.19162	1.03E-10*	−1.10811	0.000*	1.13703	3.14E-07*
cg26681822		−1.03278	0.002*	1.01637	0.114	1.03694	0.001*
cg00321644	TLR7	−1.0028	0.592	1.01247	0.020*	−1.00222	0.67
cg06896988		−1.01684	0.135	−1.0093	0.404	1.0084	0.451
cg07210784		−1.00313	0.084	1.00278	0.123	−1.00056	0.751
cg13581155		1.01088	0.61	1.01588	0.458	−1.01742	0.416
cg24735671		−1.10476	2.04E-06*	−1.065	0.001*	1.0535	0.008*
cg00741717	TLR8	−1.03908	0.001*	−1.01974	0.090	−1.00236	0.836
cg07759587		−1.18112	1.26E-06*	−1.1118	0.001*	1.09883	0.003*
cg13153942		−1.04969	0.001*	−1.00852	0.540	−1.11606	8.14E-11*
cg01395047	TLR9	1.01946	0.002*	−1.00006	0.991	−1.01477	0.015*
cg01564045		−1.01836	0.094	−1.0039	0.717	−1.01216	0.263
cg04206665		−1.00824	0.031*	−1.00954	0.013*	1.002	0.592
cg05778154		−1.00788	0.02*	1.0002	0.951	−1.0244	8.99E-10*
cg06844837		−1.00343	0.361	1.00104	0.780	−1.01657	4.51E-05*
cg14528193		−1.00564	0.078	1.0032	0.314	−1.03085	1.46E-13*
cg16302310		−1.01916	0.002*	−1.02452	0.000*	−1.00813	0.173
cg21578541		−1.05103	8.00E-07*	−1.04956	0.000*	1.04769	2.94E-06*
cg22484793		1.01453	0.057	1.01557	0.042*	−1.03657	1.10E-05*
cg06935464	TLR10	−1.00773	0.058	−1.0043	0.285	1.02615	2.31E-08*
cg17671577		−1.00355	0.204	1.00433	0.123	−1.01443	2.88E-06*
cg19398783		−1.00751	0.002*	−1.0088	0.000*	1.01243	1.51E-06*
cg22128849		−1.00075	0.665	−1.00028	0.873	−1.00119	0.494
cg23855121		−1.03164	1.37E-06*	−1.02804	0.000*	−1.00137	0.813

KD1: Kawasaki disease prior to IVIG treatment; KD1: Kawasaki disease after IVIG treatment > 3 weeks; FC: febrile control; HC: healthy control.

## DISCUSSION

To the best of our knowledge, this study is the first to comprehensively report that TLRs were considerably elevated in acute-stage KD patients and significantly decreased following IVIG administration. Although the etiology of KD remains unknown, we found that KD stimulated the extraordinary up-regulation of most TLRs, except for 3 and 7 mRNA expressions. More importantly, the mRNA levels of TLR1, 4, 5, and 8 were significantly elevated in KD patients compared to both the healthy and febrile controls. We also observed that treating KD patients with IVIG could significantly decrease these TLRs’ mRNA expression. In KD patients before undergoing IVIG treatment, TLR1, 2, 4, 6, 8, and 9 demonstrate a hypo-methylated status compared to the control subjects and KD patients after IVIG treatment. In contrast, the mRNA expression level and DNA methylation of these TLRs demonstrate an opposite reverse tendency between the two stages of KD samples and both controls.

Immune system activation is a trademark characteristic of KD [6, 17, 18]. Children with certain single nucleotide polymorphisms (SNPs) of immune genes (ex. *CD40, BLK, ITPKC, and FCGR2A*) [19, 20] are susceptible to triggering over activated inflammatory reactions through certain pathogens with a unique pathogen-associated molecular pattern (PAMP) that may be KD's immunopathogenesis. Schulte et al. have also demonstrated that both innate and adaptive immune mechanisms are critical in a murine model of coronary arteritis mimicking KD [21].

TLRs recognize a group of pathogen-associated molecular patterns but also damage the associated molecular patterns. High mobility group box 1 (HMGB1), an endogenous damage associated with molecular patterns, serves as danger signaling and provides a framework for understanding the overlapping inflammatory responses activated by pathogens and injury [22]. Furthermore, elevated HMGB1 levels have been found in KD patients [23] as a potential marker for poor response to IVIG treatment [23]. Similarly, HMGB1 has been shown not only to amplify inflammation but also to increase vascular inflammation in various vasculitis diseases [24]. The receptors involved in HMGB1 signal transduction are TLR2 and TLR4 [25], which is consistent with our findings that both TLR2 and TLR4 increased in KD patients and then decreased after IVIG treatment. Moreover, TLR4 dependent macrophage signaling is associated with coronary arterial disease [26]. Lipopolysaccharide, a TLR4 agonist, has been shown to drive hepcidin expression [27]. Hepcidin is triggered during infections and inflammation [28]. In a previous study, we were the first to show that hepcidin induces transient anemia and hypoferremia in the acute inflammatory phase of KD [29]. Furthermore, TLR6 functionally produces heterodimer with TLR2 to facilitate intracellular response to bacterial lipoprotein [30], while TLR9 appears to respond to unmethylated CpG of bacterial DNA [8]. Our results showed remarkable activation of TLR1, 2, 4, 5, 6, and 9, which correlates with bacteria-related pathogen-associated molecular patterns, except for activation of TLR3 and 7, which relates to double-stranded RNA and single-stranded viral RNA in the acute stage of KD. These results support the evidence that KD is a bacterium-like inflammatory disease. During the past 50 years, since Dr. Kawasaki's first report in 1967, many studies have attempted to identify a definitive infectious agent that causes KD but have not succeeded. However, certain superantigens from bacteria and intracellular pathogens have been implicated in KD. Various bacteria, including *Streptococcus pyogenes*, *Staphylococcus aureus*, *Mycoplasma pneumoniae*, *Yersinia pseudotuberculosis*, and *Chlamydia pneumoniae*, have been isolated in KD patients [31, 32]. On the other hand, Chang et al. reported that common respiratory viruses, such as coronaviruses, adenoviruses, enteroviruses, and rhinoviruses, were associated with KD [33]. Stating for certain whether KD is the result of a bacterial infection or a viral infection is still not possible. Taken together, KD is not a purely infectious disease with a cause-effect relationship with any pathogens but can occur in hosts with certain genetic backgrounds.

Since IVIG is commonly understood to exert anti-inflammatory properties in KD, we also reported that IVIG treatment has a better impact on global methylation alteration compared to KD's onset [15]. In this study, we demonstrated the DNA methylation on TLRs and alterations between KD patients before and after IVIG administration and the control subjects. We then showed that hypomethylation at gene promoters of TLR1, 2, 4, 6, 8, and 9 increases the expression of downstream genes. Vogelpoel et al. reported that the cross-talk between *FCGR2A* and TLRs in human dendritic cells activated anti-bacterial immunity through the selective induction of TNFα and Th17-promoting cytokines response, which agrees with our previous findings regarding Th-17, TNFα, and FCGR2A in KD [17, 34]. The present study is the first to demonstrate that epigenetics of DNA methylation on TLRs may influence downstream gene expression in KD.

## MATERIALS AND METHODS

### Patients

This study consists of a total of 54 subjects from Kaohsiung Chang Gung Memorial Children's Hospital in Taiwan from 2012 to 2014. Of those, 18 subjects were healthy controls (with neither fever nor KD history), 18 subjects were fever controls (with fever but not diagnosed to have KD), and the remaining 18 subjects were KD patients that met the KD diagnosis criteria and had samples collected both before and after IVIG treatment [35, 36] administered at the hospital. The fever control group included patients that had been admitted to the hospital with an acute infection, including acute pharyngitis, acute tonsillitis, croup, acute bronchitis, pyuria, and acute bronchiolitis. Peripheral blood samples of KD patients were taken twice: once prior to receiving IVIG treatment (pre-IVIG) and once at least three weeks after completing IVIG treatment, with the latter functioning as the convalescent stage samples (post-IVIG > 3 weeks) [15]. The Chang Gung Memorial Hospital's Institutional Review Board (IRB No.:102-3779A3) approved this study, and we obtained the written informed consent from the parents or guardians of all the subjects. All of the methods used comply with the relevant approved guidelines.

### Experiment design

First, we collected whole blood samples from the subjects, followed by white blood cell (WBC) enrichment. The enriched WBC samples were subjected to either RNA or DNA extraction. Pursuant to the manufacturer's instructions, we used an isolation kit (mirVana™ miRNA Isolation Kit, Catalog number: AM1560, Life Technologies, Carlsbad, CA) to isolate total RNA. Then we measured the collected RNA samples with Bioanalyzer (ABI) and Qubit (Thermo) to determine RNA quality (RIN value) and quantity. All RNA samples passed the criterion of RIN ≥ 7. The DNA samples were isolated and treated with bisulfite as described in a previous study [37].

### Gene expression profiling with microarray

For strong, unbiased results, pooled RNA libraries were produced by evenly pooling six RNA samples, which resulted in three pooled normal control, three fever control, three pre-IVIG, and three post-IVIG libraries. The pooled RNA samples underwent microarray assay to determine gene expression profile. In this study, we carried out profiling with GeneChip^®^ Human Transcriptome Array 2.0 (HTA 2.0, Affymetrix, Santa Clara). The RNA samples were prepared using the WT PLUS Reagent kit, followed by hybridization on HTA 2.0 microarray chips. In accordance with Affymetrix manuals, the raw data of the HTA 2.0 chips underwent quality control examination. The chips that passed the quality control criteria were analyzed with Partek (Partek, St. Louis), a commercial software specifically for microarray data analysis. Using Partek, we conducted ANOVA analysis and reported the *p*-values of the comparisons of interest.

### DNA methylation profiling with Illumina M450K BeadChip

We used Illumina HumanMethylation450 (M450K) BeadChip to perform genome-wide screening of DNA methylation patterns. M450K BeadChip was designed to detect methylation patterns of approximately 450,000 CpG markers that span the human genome. More information about M450 BeadChip can be found at http://support.illumina.com/array/array_kits/infinium_humanmethylation450_beadchip_kit.html For each M450K BeadChip assay, 200 ng of bisulfite-converted genomic DNA was applied in accordance with the manufacturer's instructions. Afterward, for each CpG marker in each sample, we determined the methylation percentage of cytosine, referred to as β values.

### Statistical analysis

All data are presented as mean ± standard error. We applied Student's *t*-test or, when appropriate, one-way ANOVA to analyze quantitative data. We evaluated any data changes before and after IVIG treatment using the paired sample *t*-test. We performed all statistical analyses using SPSS version 22.0 for Windows XP (SPSS, Inc., Chicago, USA), and a two-sided *p*-value less than 0.05 is considered statistically significant.

## CONCLUSIONS

KD patients showed remarkably increased TLRs’ mRNA expression and hypomethylation at the gene promoters of TLRs. Meanwhile, treating KD patients with IVIG can significantly decrease these TLRs’ mRNA expression and restore the methylation of TLRs. TLRs, especially TLR 1, 2, 4, 6, 8, and 9, may participate in KD's immunopathogenesis.

## References

[1] Kuo HC, Hsu YW, Wu MS, Chien SC, Liu SF, Chang WC (2016). Intravenous immunoglobulin, pharmacogenomics, and Kawasaki disease. J Microbiol Immunol Infect.

[2] Newburger JW, Takahashi M, Burns JC, Beiser AS, Chung KJ, Duffy CE, Glode MP, Mason WH, Reddy V, Sanders SP (1986). The treatment of Kawasaki syndrome with intravenous gamma globulin. N Engl J Med.

[3] Brown RL, Clarke TB (2017). The regulation of host defences to infection by the microbiota. Immunology.

[4] Chen K, Huang J, Gong W, Iribarren P, Dunlop NM, Wang JM (2007). Toll-like receptors in inflammation, infection and cancer. Int Immunopharmacol.

[5] Lin IC, Kuo HC, Lin YJ, Wang FS, Wang L, Huang SC, Chien SJ, Huang CF, Wang CL, Yu HR, Chen RF, Yang KD (2012). Augmented TLR2 expression on monocytes in both human Kawasaki disease and a mouse model of coronary arteritis. PLoS One.

[6] Kuo HC, Yang YL, Chuang JH, Tiao MM, Yu HR, Huang LT, Yang KD, Chang WC, Lee CP, Huang YH (2012). Inflammation-induced hepcidin is associated with the development of anemia and coronary artery lesions in Kawasaki disease. J Clin Immunol.

[7] Weng KP, Ho TY, Chiao YH, Cheng JT, Hsieh KS, Huang SH, Ou SF, Liu KH, Hsu CJ, Lu PJ, Hsiao M, Ger LP (2010). Cytokine genetic polymorphisms and susceptibility to Kawasaki disease in Taiwanese children. Circ J.

[8] Leifer CA, Medvedev AE (2016). Molecular mechanisms of regulation of Toll-like receptor signaling. J Leukoc Biol.

[9] Principi N, Rigante D, Esposito S (2013). The role of infection in Kawasaki syndrome. J Infect.

[10] Katano H, Sato S, Sekizuka T, Kinumaki A, Fukumoto H, Sato Y, Hasegawa H, Morikawa S, Saijo M, Mizutani T, Kuroda M (2012). Pathogenic characterization of a cervical lymph node derived from a patient with Kawasaki disease. Int J Clin Exp Pathol.

[11] Rosenkranz ME, Schulte DJ, Agle LM, Wong MH, Zhang W, Ivashkiv L, Doherty TM, Fishbein MC, Lehman TJ, Michelsen KS, Arditi M (2005). TLR2 and MyD88 contribute to Lactobacillus casei extract-induced focal coronary arteritis in a mouse model of Kawasaki disease. Circulation.

[12] Wang GB, Li CR, Zu Y, Yuan XW (2006). [The role of activation of toll-like receptors in immunological pathogenesis of Kawasaki disease]. Zhonghua Er Ke Za Zhi.

[13] Mortazavi SH, Amin R, Alyasin S, Kashef S, Karimi MH, Babaei M, Younesi V (2015). Down-regulation of TLR2, 3, 9 and Signaling Mediators, MyD88 and TRIF, Gene Transcript Levels in Patients with Kawasaki Disease Treated with IVIG. Iran J Allergy Asthma Immunol.

[14] Wu H, Zhang Y (2014). Reversing DNA methylation: mechanisms, genomics, and biological functions. Cell.

[15] Li SC, Chan WC, Huang YH, Guo MM, Yu HR, Huang FC, Kuo HC, Kuo HC (2016). Major methylation alterations on the CpG markers of inflammatory immune associated genes after IVIG treatment in Kawasaki disease. BMC medical genomics.

[16] Tsai KW, Chang B, Pan CT, Lin WC, Chen TW, Li SC (2015). Evaluation and application of the strand-specific protocol for next-generation sequencing. Biomed Res Int.

[17] Guo MM, Tseng WN, Ko CH, Pan HM, Hsieh KS, Kuo HC (2015). Th17- and Treg-related cytokine and mRNA expression are associated with acute and resolving Kawasaki disease. Allergy.

[18] Huang YH, Hsu YW, Lu HF, Wong HS, Yu HR, Kuo HC, Huang FC, Chang WC, Kuo HC (2016). Interferon-gamma Genetic Polymorphism and Expression in Kawasaki Disease. Medicine.

[19] Lee YC, Kuo HC, Chang JS, Chang LY, Huang LM, Chen MR, Liang CD, Chi H, Huang FY, Lee ML, Huang YC, Hwang B, Chiu NC (2012). Two new susceptibility loci for Kawasaki disease identified through genome-wide association analysis. Nat Genet.

[20] Kuo HC, Yang KD, Juo SHH, Liang CD, Chen WC, Wang YS, Lee CH, Hsi E, Yu HR, Woon PY, Lin IC, Huang CF, Hwang DY (2011). ITPKC single nucleotide polymorphism associated with the Kawasaki disease in a Taiwanese population. PLoS One.

[21] Schulte DJ, Yilmaz A, Shimada K, Fishbein MC, Lowe EL, Chen S, Wong M, Doherty TM, Lehman T, Crother TR, Sorrentino R, Arditi M (2009). Involvement of innate and adaptive immunity in a murine model of coronary arteritis mimicking Kawasaki disease. J Immunol.

[22] Ellerman JE, Brown CK, de Vera M, Zeh HJ, Billiar T, Rubartelli A, Lotze MT (2007). Masquerader: high mobility group box-1 and cancer. Clin Cancer Res.

[23] Eguchi T, Nomura Y, Hashiguchi T, Masuda K, Arata M, Hazeki D, Ueno K, Nishi J, Kawano Y, Maruyama I (2009). An elevated value of high mobility group box 1 is a potential marker for poor response to high-dose of intravenous immunoglobulin treatment in patients with Kawasaki syndrome. Pediatr Infect Dis J.

[24] Wang C, de Souza AW, Westra J, Bijl M, Chen M, Zhao MH, Kallenberg CG (2015). Emerging role of high mobility group box 1 in ANCA-associated vasculitis. Autoimmunity reviews.

[25] Bianchi ME, Manfredi AA (2007). High-mobility group box 1 (HMGB1) protein at the crossroads between innate and adaptive immunity. Immunological reviews.

[26] Habib A, Polavarapu R, Karmali V, Guo L, Van Dam R, Cheng Q, Akahori H, Saeed O, Nakano M, Pachura K, Hong CC, Shin E, Kolodgie F (2015). Hepcidin-ferroportin axis controls toll-like receptor 4 dependent macrophage inflammatory responses in human atherosclerotic plaques. Atherosclerosis.

[27] Pietrangelo A, Dierssen U, Valli L, Garuti C, Rump A, Corradini E, Ernst M, Klein C, Trautwein C (2007). STAT3 is required for IL-6-gp130-dependent activation of hepcidin in vivo. Gastroenterology.

[28] Le NT, Richardson DR (2002). Ferroportin1: a new iron export molecule?. Int J Biochem Cell Biol.

[29] Huang YH, Kuo HC, Huang FC, Yu HR, Hsieh KS, Yang YL, Sheen JM, Li SC, Kuo HC (2016). Hepcidin-Induced Iron Deficiency Is Related to Transient Anemia and Hypoferremia in Kawasaki Disease Patients. Int J Mol Sci.

[30] Noreen M, Arshad M (2015). Association of TLR1, TLR2, TLR4, TLR6, and TIRAP polymorphisms with disease susceptibility. Immunol Res.

[31] Kusuda T, Nakashima Y, Murata K, Kanno S, Nishio H, Saito M, Tanaka T, Yamamura K, Sakai Y, Takada H, Miyamoto T, Mizuno Y, Ouchi K (2014). Kawasaki disease-specific molecules in the sera are linked to microbe-associated molecular patterns in the biofilms. PLoS One.

[32] Wang CL, Wu YT, Liu CA, Kuo HC, Yang KD (2005). Kawasaki disease: infection, immunity and genetics. Pediatr Infect Dis J.

[33] Chang LY, Lu CY, Shao PL, Lee PI, Lin MT, Fan TY, Cheng AL, Lee WL, Hu JJ, Yeh SJ, Chang CC, Chiang BL, Wu MH, Huang LM (2014). Viral infections associated with Kawasaki disease. J Formos Med Assoc.

[34] Vogelpoel LT, Hansen IS, Visser MW, Nagelkerke SQ, Kuijpers TW, Kapsenberg ML, de Jong EC, den Dunnen J (2015). FcgammaRIIa cross-talk with TLRs, IL-1R, and IFNgammaR selectively modulates cytokine production in human myeloid cells. Immunobiology.

[35] Newburger JW, Takahashi M, Gerber MA, Gewitz MH, Tani LY, Burns JC, Shulman ST, Bolger AF, Ferrieri P, Baltimore RS, Wilson WR, Baddour LM, Levison ME (2004). Diagnosis, treatment, and long-term management of Kawasaki disease: a statement for health professionals from the Committee on Rheumatic Fever, Endocarditis and Kawasaki Disease, Council on Cardiovascular Disease in the Young, American Heart Association. Circulation.

[36] Kuo HC, Lo MH, Hsieh KS, Guo MM, Huang YH (2015). High-Dose Aspirin Is Associated with Anemia and Does Not Confer Benefit to Disease Outcomes in Kawasaki Disease. PLoS One.

[37] Kuo HC, Chang JC, Yu HR, Wang CL, Lee CP, Huang LT, Yang KD (2015). Identification of an association between genomic hypomethylation of FCGR2A and susceptibility to Kawasaki disease and intravenous immunoglobulin resistance by DNA methylation array. Arthritis Rheumatol.

